# Improving the sensitivity of myelin oligodendrocyte glycoprotein-antibody testing: exclusive or predominant MOG-IgG3 seropositivity—a potential diagnostic pitfall in patients with MOG-EM/MOGAD

**DOI:** 10.1007/s00415-024-12285-5

**Published:** 2024-04-13

**Authors:** S. Jarius, M. Ringelstein, K. Schanda, K. Ruprecht, M. Korporal-Kuhnke, A. Viehöver, M. W. Hümmert, P. Schindler, V. Endmayr, M. Gastaldi, C. Trebst, D. Franciotta, O. Aktas, R. Höftberger, J. Haas, L. Komorowski, F. Paul, M. Reindl, B. Wildemann

**Affiliations:** 1 Molecular Neuroimmunology Group, Department of Neurology, University of Heidelberg, Heidelberg, Germany; 2 Department of Neurology, Heinrich Heine University, Düsseldorf, Germany; 3 Clinical Department of Neurology, Medical University of Innsbruck, Innsbruck, Austria; 4 Department of Neurology, Charité – Universitätsmedizin Berlin, corporate member of Freie Universität Berlin and Humboldt-Universität zu Berlin, Berlin, Germany; 5 Department of Neurology, Hannover Medical School, Hanover, Germany; 6 Division of Neuropathology and Neurochemistry, Department of Neurology, Medical University of Vienna, Vienna, Austria; 7 Comprehensive Center for Clinical Neurosciences and Mental Health, Medical University of Vienna, Vienna, Austria; 8 Neuroimmunology Laboratory and Neuroimmunology Research Unit, IRCCS Mondino Foundation National Neurological Institute, Pavia, Italy; 9 Institute of Experimental Neuroimmunology, affiliated to Euroimmun AG, Lübeck, Germany; 10 Experimental and Clinical Research Center, a cooperation between the Max Delbrück Center for Molecular Medicine in the Helmholtz Association and Charité – Universitätsmedizin Berlin, Berlin, Germany; 11 Neuroscience Clinical Research Center, Charité – Universitätsmedizin Berlin, corporate member of Freie Universität Berlin and Humboldt-Universität zu Berlin, and Berlin Institute of Health, Berlin, Germany

**Keywords:** Myelin oligodendrocyte glycoprotein (MOG), MOG antibody-associated encephalomyelitis (MOG-EM), MOG antibody-associated disease (MOGAD), Multiple sclerosis, Neuromyelitis optica spectrum disorders (NMOSD), Optic neuritis, Myelitis, Immunoglobulin G (IgG), IgG subclasses, MOG-IgG1, MOG-IgG3, Seroreversion, Seroconversion, Seronegativity, Seronegative, Aquaporin-4 (AQP4), Tests, Assays, Serology, Autoantibody, Antibodies, Detection antibodies, Sensitivity, Rozanolixizumab

## Abstract

**Background:**

Myelin oligodendrocyte glycoprotein antibody-associated encephalomyelitis (MOG-EM; also termed MOG antibody-associated disease, MOGAD) is the most important differential diagnosis of both multiple sclerosis and neuromyelitis optica spectrum disorders. A recent proposal for new diagnostic criteria for MOG-EM/MOGAD explicitly recommends the use of immunoglobulin G subclass 1 (IgG1)- or IgG crystallizable fragment (Fc) region-specific assays and allows the use of heavy-and-light-chain-(H+L) specific assays for detecting MOG-IgG. By contrast, the utility of MOG-IgG3-specific testing has not been systematically evaluated.

**Objective:**

To assess whether the use of MOG-IgG3-specific testing can improve the sensitivity of MOG-IgG testing.

**Methods:**

Re-testing of 22 patients with a definite diagnosis of MOG-EM/MOGAD and clearly positive MOG-IgG status initially but negative or equivocal results in H+L- or Fc-specific routine assays later in the disease course (i.e. patients with spontaneous or treatment-driven seroreversion).

**Results:**

In accordance with previous studies that had used MOG-IgG1-specific assays, IgG subclass-specific testing yielded a higher sensitivity than testing by non-subclass-specific assays. Using subclass-specific secondary antibodies, 26/27 supposedly seroreverted samples were still clearly positive for MOG-IgG, with MOG-IgG1 being the most frequently detected subclass (25/27 [93%] samples). However, also MOG-IgG3 was detected in 14/27 (52%) samples (from 12/22 [55%] patients). Most strikingly, MOG-IgG3 was the predominant subclass in 8/27 (30%) samples (from 7/22 [32%] patients), with no unequivocal MOG-IgG1 signal in 2 and only a very weak concomitant MOG-IgG1 signal in the other six samples. By contrast, no significant MOG-IgG3 reactivity was seen in 60 control samples (from 42 healthy individuals and 18 patients with MS). Of note, MOG-IgG3 was also detected in the only patient in our cohort previously diagnosed with MOG-IgA^+^/IgG^–^ MOG-EM/MOGAD, a recently described new disease subvariant. MOG-IgA and MOG-IgM were negative in all other patients tested.

**Conclusions:**

In some patients with MOG-EM/MOGAD, MOG-IgG is either exclusively or predominantly MOG-IgG3. Thus, the use of IgG1-specific assays might only partly overcome the current limitations of MOG-IgG testing and—just like H+L- and Fcγ-specific testing—might overlook some genuinely seropositive patients. This would have potentially significant consequences for the management of patients with MOG-EM/MOGAD. Given that IgG3 chiefly detects proteins and is a strong activator of complement and other effector mechanisms, MOG-IgG3 may be involved in the immunopathogenesis of MOG-EM/MOGAD. Studies on the frequency and dynamics as well as the clinical and therapeutic significance of MOG-IgG3 seropositivity are warranted.

**Supplementary Information:**

The online version contains supplementary material available at 10.1007/s00415-024-12285-5.

## Dear Sirs,

Myelin oligodendrocyte glycoprotein antibody-associated encephalomyelitis (MOG-EM; also termed MOG antibody-associated disease, MOGAD) has recently been established to be one of the most important differential diagnoses of multiple sclerosis (MS), aquaporin-4 (AQP4) immunoglobulin (Ig) G-seropositive neuromyelitis optica spectrum disorders (NMOSD), and a number of other inflammatory disorders of the central nervous system (CNS) [10, 12, 20, 23]. The diagnosis of MOG-EM/MOGAD is defined by the presence of certain clinical and radiological features in association with autoantibodies to MOG [2, 10]) and, thus, depends strongly on the use of reliable serological assays. However, no generally accepted gold standard assay exists thus far. Instead, a multitude of different assays are currently in use worldwide. Moreover, testing for MOG-IgG has been found to be methodologically much more challenging than testing for antibodies to AQP4, the main target antigen in NMOSD [19]. This is reflected by a significant level of discordance between assays and laboratories, especially when it comes to low-titre or borderline results. Differences in transfection rates and methods, fixation (formalin vs. no fixation) and the detection antibodies used (heavy and light [H+L] chain-specific vs. Fc/Fcγ-specific vs. IgG1-specific) are thought to underlie some of the issues currently associated with MOG-IgG serology. False-negative test results may easily translate into false treatment decisions, however, with undesirable consequences for the patients affected.

Here, we would like to point to a potentially important diagnostic pitfall, namely false-negative MOG-IgG testing due to the presence of exclusively or predominantly MOG-IgG3. This may result in underdiagnosis of MOG-EM/MOGAD as well as in premature suspension of long-term immunotherapy due to supposed disappearance of the antibody. Human IgG3 is considered an “understudied but highly potent immunoglobulin” [3].

In our index sample (sample 1A; see Table 1), from a patient with bilateral optic neuritis and longitudinally extensive transverse myelitis (negative oligoclonal bands in the cerebrospinal fluid [CSF], > 100 cells/µl CSF), a positive MOG-IgG result was obtained on testing by means of a fixed, Fcγ-specific cell-based assay (CBA) [15, 18] (Table 1). As the positive signal was very weak, we tested the sample in a second assay employing IgG subclass-specific detection antibodies (Binding Site, Schwetzingen, Germany) [17]. In this assay, the sample was clearly positive for MOG-IgG, with, surprisingly, IgG3 being the predominant subclass (semiquantitative signal intensity at standard starting dilution: IgG3 + + + +  >>> IgG1 +, IgG2 +, IgG4 +; end titres: IgG3 ≥ 1:320, IgG1 1:10, IgG2 1:10, IgG4 1:10 [cut-off ≥ 1:10]) (Supplementary Figure). In addition, MOG-IgA was positive (+ + + +); MOG-IgM was negative.

**Table 1:** MOG-IgG, -IgG1, -IgG2, -IgG3, -IgG4, -IgM and -IgA results in 27 samples from 22 patients with supposed seroreversion to negative according to at least one routine CBA test (24 × H+L-negative, 18 × Fc-negative)

Pat.no./sex/clinical findings	Fixed CBA1 IgG (Fcγ)	Fixed CBA2 IgG (Fcγ)	Live CBA1 IgG (H+L)	Live CBA2 IgG (Fc)	Live CBA3 IgG (H+L)	Fixed CBA1 IgG1	Fixed CBA1 IgG2	Fixed CBA1 IgG3	Fixed CBA1 IgG4	Fixed CBA1 IgA	Fixed CBA1 IgM	MOG-IgG in other samples from the same patient
#1A/f/bON+LETM	2 × +	“Neg”	Neg	N.d	Neg (1:80)	+ weak	+	**+ + + + **(> 1:320)	+ weak	+ + + +	–	IgG neg, IgA pos (Fc-fixed; pos in several samples)
#1B/f/bON+LETM	2 × +	“Neg”	Neg (1:40)	N.d	Neg (1:80)	–	+	**+ + + + **(> 1:320)	+ weak	+ + +	–	
#2/m/rON	+	Neg	Neg (1:80)	N.d	Neg (1:80)	+	–	**+ + + **	–	–	–	1:320 (H+L), + (Fc-fixed)
#3/f/LETM	2 × neg	Neg	Neg (1:80)	N.d	Neg	+	–	**+ + + + **	–	–	–	1:320 (H+L1), + (Fc-fixed), “pos” (H+L, external lab)
#4/m/NETM	1 × +, 1 × +/–	“Pos”	Neg (1:40)	Pos (1:160)	Neg	+	+ +	**+ + + + + **	–	–	–	“Pos” (Fc-fixed2; confirmed in 4 further samples)
#5/f/BST	+ + +	“Pos 1:32”	Neg (1:80)	Pos (1:160)	N.d	+	+	**+ + + + + **	+/﻿–	–	–	1:320 (Fc-live), 1:160 (H+L), + + (Fc-fixed1), “Pos 1:100” (Fc-fixed2)
#6/f/NMOSD	1 × + weak, 1 × +/–	Neg	Neg (1:80)	N.d	N.d	+	N.d	**+ + + **	N.d	N.d	N.d	1:320 (H+L), pos (Fc-fixed1)
#7/f/LETM	2 × neg	Neg	1 × pos (1:160), 1 × neg (1:80)	Neg (1:80)	Neg	1 × +/–, 1× neg	+/-	**+ + **	–	–	–	“Pos” (live CBA)
#8A/f/ON+LETM	+/–	“Neg”	Neg (1:80)	Pos (1:160)	Neg (1:80)	+ + +	–	+ +	–	–	–	1:5120 (H+L), + + + + + (Fc-fixed1); 3 further samples pos (Fc-fixed1+2)
#8B/f/ON+LETM	Neg	Neg	Pos (1:160)	Pos (1:320)	Neg (1:80)	+ + + +	–	–	–	–	–	1:5120 (H+L), + + + + + (Fc-fixed1); 3 further samples pos (Fc-fixed1+2)
#9/f/rON+LETM	+/– str.bg	“Pos 1:10”	Neg (1:80)	N.d	Pos (1:320)	+	+/–	+	–	–	–	1:320 (H+L), + + (Fc-fixed)
#10/f/ON+LETM	1 × +/–, 1 × neg	Neg	Pos (1:320)	N.d	Neg (1:80)	+ + + +	–	+	–	– (2 ×)	–	1:320 (H+L), +/– (Fc-fixed), 2 × neg (Fc-fixed)
#11f/rON	+ +	N.d	1 × pos (1:160), 1 × neg (1:40)	N.d	Neg (1:80)	+ + +	+	–	–	–	–	1:320 (H+L), + + + (Fc-fixed)
#12/m/ON+BST	Neg	Neg	Neg (1:80)	Pos (1:160)	Neg (1:40)	+ +	–	–	–	+/﻿–	–	1:640 (H + L), + + (Fc-fixed), “1:320 (live CBA)”
#13/f/LETM	1 × +/–, 1 × neg	“Pos 1:10”	Neg (1:80)	Pos (1:160)	N.d	+ + + +	+ +	–	–	–	–	> 1:320 (Fc-live), 1:160 (H+L), pos (Fc-fixed2)
#14/f/ON	+ + +	N.d	Neg (1:80)	Pos (1:160)	N.d	+ + + + +	+ +	–	–	–	–	1:320 (Fc-live), 1:160 (H+L), pos + + + + (Fc-fixed1)
#15/f/ON+MY+BST	1 × + + +, 1 × + +	N.d	Neg (1:80)	Pos (1:160)	N.d	+ + + + +	+ +	+ +	+	–	–	1:320, 1:160 (Fc-live), 1:320, 1:160 (H+L), + + + (5 ×; Fc-fixed1), pos (Fc-fixed2)
#16/f/rON	1 × + + +, 1 × + + + +	Pos (1:32)	1 × pos (1:320), 1 × neg (1:80)	N.d	Neg (1:40)	+ + + +	+ + +	–	+/﻿–	–	–	“Pos 1:32”, 3 × ”pos 1:10″ (Fc-fixed)
#17/f/ON+LETM	+ + +	N.d	Neg (1:80)	N.d	N.d	+ + + +	N.d	+/﻿–	N.d	–	–	1:2560, 1:1280, 1:160 (H+L), + + + + + (Fc-fixed1), pos (Fc-fixed2; 4 samples)
#18/f/rON	+ + + +	Pos (1:32)	Neg (1:40)	N.d	N.d	+ + + + +	N.d	+ +	N.d	N.d	N.d	1:320 (H+L; 6 samples), + + + + (Fc-fixed)
#19A/m/LETM	+ +	Neg	Neg (1:40)	N.d	Neg (1:20)	+ + +	–	+/﻿–	–	–	+/﻿–	1:640, 1:160 (H+L), + + (Fc-fixed)
#19B/m/ON+LETM	+ weak	Neg	Neg (1:80)	N.d	N.d	+ + +	N.d	–	N.d	N.d	N.d	1:640, 1:160 (H+L), + + (Fc-fixed)
#19C/m/ON+LETM	Neg	Neg	Neg (1:40)	N.d	N.d	+ +	N.d	–	N.d	N.d	N.d	1:640, 1:160 (H+L), + + (Fc-fixed)
#20A/f/LETM	+ + +	“Pos 1:100”	Neg (1:80)	N.d	N.d	+ + + +	N.d	+/﻿–	N.d	N.d	N.d	1:2560, 1:1280, 1:160 (H+L), 2 × + + + (Fc-fixed)
#20B/f/LETM	Neg	“Neg”	Neg (1:40)	N.d	N.d	+ +	N.d	+/﻿–	N.d	N.d	N.d	1:2560, 1:1280, 1:160 (H+L), 2 × + + + (Fc-fixed)
#21/f/rON	+ + +	N.d	Neg (1:80)	Pos (1:160)	N.d	+ + + +	N.d	+ +	N.d	N.d	N.d	1:1280, 1:320, 1:160 (H+L); 1:320 (Fc-live), + + + (Fc-fixed)
#22/m/ADEM	Neg	Neg	Neg (1:80)	Neg (1:80)	N.d	+ +	N.d	–	N.d	N.d	N.d	Pos CSF (1:16, H+L; 1:64 Fc-live; serum not available)

All samples were obtained from patients that had been unequivocally positive for MOG antibodies in earlier samples (see last column for details). Note that cut-offs vary between the various assays (fixed CBAs: ≥ 1:10; live CBAs: ≥ 1:160). Signal intensity at 1:10 starting dilution was semi-quantitatively categorized in the fixed CBAs (+ very weak to + + + + + very strong; NB: a very weakly positive [+] IgG1 and IgG3 signal was also observed with 1 out of 60 control samples; +/﻿– denotes equivocal results, not classified as positive). Results set in quotation marks were taken from the patient records.

*ADEM* acute disseminated encephalomyelitis, *bON* bilateral ON, *BST* brainstem encephalitis, *CBA* cell-based assay, *Fc-fixed/live* fixed/live CBA employing a Fc- or Fcγ-specific detection antibody (Fc-fixed 1: in-house, University of Heidelberg, Germany; *Fc-fixed 2*: external laboratory), *Fc/Fcγ*, Fc/Fcγ fragment-specific detection antibody; *HEK293*, human embryonic kidney 293 cells; *H+L*, live CBA employing a heavy and light chain-specific detection antibody (H+L1: laboratory 1; H+L2: laboratory 2); *LETM*, longitudinally extensive transverse myelitis; *n.a.*, not available; *n.d.*, not done;* neg*, negative;* NETM*, non-longitudinally extensive transverse myelitis; *NMOSD*, neuromyelitis optica spectrum disorder; *ON*, optic neuritis; *PA*, after precipitation (i.e. elimination) of total IgG (see ref. [9] for methods); *pos*, positive; *rON*, recurrent ON; *str.bg.* = strong background staining

A follow-up sample (sample 1B; see Table 1) from the same patient, obtained around 2 years later, again yielded only a very weak signal in the Fcγ-specific fixed MOG-CBA. While there was no longer a clearly positive MOG-IgG1 signal, MOG-IgG3 was still strongly detectable (Supplementary Figure and Table 1). MOG-IgA was again positive and MOG-IgG negative. The two samples, originally obtained 2 years apart, were tested side-by-side using the same batch of MOG-transfected cells.

Of note, both samples (1A and 1B) had previously tested negative for total MOG-IgG in three live CBAs employing H+L- or Fc fragment-specific detection antibodies, respectively, with a titre just below the cut-off (maximum titre 1:80; cut-off of 1:160), as well as in an Fcγ-specific commercial assay [13] (Table 1).

Given the surprising finding of predominant MOG-IgG3 seropositivity in this patient and considering previous reports suggesting favourable sensitivity of IgG1-specific MOG assays relative to standard assays, we decided to investigate whether the sensitivity of MOG-IgG testing could be generally improved by implementing MOG-IgG3-specific testing.

To address this question, we tested 25 additional serum samples from 21 further patients. All patients met the following criteria: (a) previous diagnosis of MOG-EM/MOGAD based on the presence of an unequivocally positive MOG-IgG titre in the past and clinico-radiological features typical for MOG-EM/MOGAD (optic neuritis, myelitis, brainstem encephalitis, acute disseminated encephalomyelitis [ADEM], AQP4-IgG-negative NMOSD) [10, 11]; (b) availability of at least one follow-up sample that had turned negative later in the disease course in at least one H+L- or Fc-specific assay (Table 1).

Strikingly, 12 (48%) of these 25 follow-up samples negative in at least one H+L- or Fc-specific assay were also positive for MOG-IgG3 (Table 1), summing up to 14 (52%) MOG-IgG3-positive samples among 27 samples tested overall (or 13 of 22 patients overall) in the total cohort (i.e. if the two index samples are counted as well). Even more importantly (from a diagnostic point of view), MOG-IgG3 was the predominant subclass in 8/27 (30%) samples (from 7 [32%] patients), with no unequivocal MOG-IgG1 signal at all in 2 of these 8 samples and only a very weak concomitant MOG-IgG1 signal in the other 6 (Table 1 and Fig. ﻿1), i.e. in 8/14 (57%) of the MOG-IgG3-positive samples. By contrast, only one sample (from a *bona fide* healthy subject; negative in an H+L-specific live CBA) out of 60 control samples (from 42 healthy individuals and 18 patients with MS) showed very weak IgG3 staining. All other controls showed no IgG3 signal at all (Table 1). In a more conservative analysis, i.e. if samples that showed only a very weak signal (indicated by “+” in Table 1), as observed with that single control, were not classified as positive for the purpose of this analysis, still 12/27 (44%) samples (from 11 patients) were positive for MOG-IgG3, 8 (66%) of which (from 7 patients) showed no concomitant MOG-IgG1 reaction (corresponding to 18 MOG-IgG1 samples in this analysis among the 27 patients tested).

**Fig. 1:**
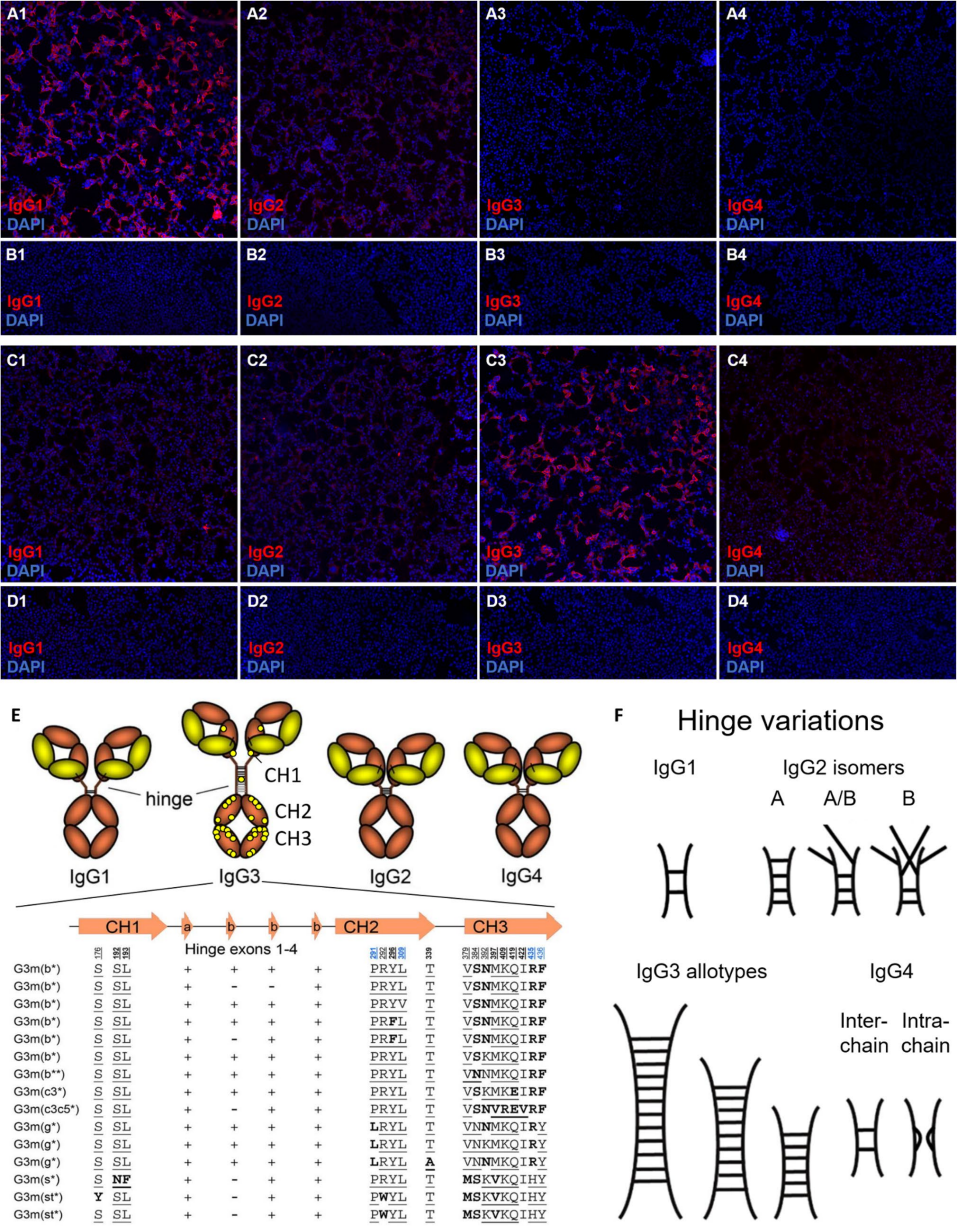
MOG-IgG1-4 subclass analysis in samples from two exemplary patients (**A**–**D**) and schematic depiction of structural differences between human IgG subclasses and IgG3 allotypes (**E**–**F**). Panels** A**-**D**: Human embryonic HEK293 cells transfected with human full-length MOG (**A**, **C**) and empty vector-transfected HEK239 control cells (**B**, **D**) were employed as test substrates. While the first patient (#13; A1-4 and B1-4) showed IgG1-dominant binding of IgG to the MOG-transfected cells (A1), the second patient (#5; C-14, D1-4) displayed an IgG3-dominant reaction (C3). Both patients had been previously clearly positive for total MOG-IgG in an H+L-specific assay but had turned negative in the same assay in the two samples shown here. In addition, a mild IgG2 co-reaction was seen in the first patient (A2) and a very mild IgG1 and IgG2 co-reaction in the second patient (C1, C2); no binding of IgG1, IgG2, IgG3 or IgG3 to any of the control substrates was observed (B1-4, D1-4). Red colour indicates binding of patient IgG of the respective IgG subclass to cells. Affinity-purified sheep anti-human IgG antibodies specific for the IgG subclasses 1, 2, 3 and 4 (Binding Site GmbH, Schwetzingen, Germany), respectively, were used to mark bound human IgG. To rule out non-specific signals, (a) an AF568-labelled donkey anti-sheep IgG antibody cross-absorbed both against human IgG and against human sera (Invitrogen, Carlsbad, USA) was used as tertiary antibody to visualize bound human IgG; (b) control experiments were performed using only the tertiary antibody or the patient’s serum and the tertiary antibody resulting in complete loss of signal (not shown). DAPI was used to stain cell nuclei (blue). Panel** E**-**F**: Schematic representation of structural differences between human IgG subclasses and IgG3 allotypes and overview of differences in hinge exons and amino acid sequence between IgG3 allotypes (modified from Vidarsson et al. Frontiers in Neurology 2014 [31], Fig. 3, and de Taeye et al. Antibodies 2019 [5], Fig. 1, under the terms of the Creative Commons Attribution License [http://creativecommons.org/licenses/by/4.0]). Amino acids printed in bold are unique for the respective allotype; those underlined characterize isoallotypes. Yellow dots indicate sites of IgG3 allotype-specific primary sequence variations. Abbreviations:* AF*, AlexaFluor^®^;* CH*, Constant heavy chain domain;* DAPI*, 4ʹ,6-diamidino-2-phenylindole;* HEK293*, human embryonic kidney 293 cells;* IgG*, immunoglobulin G;* MOG*, myelin oligodendrocyte glycoprotein

None of the 18 follow-up samples tested for all four subclasses was exclusively or predominantly positive for MOG-IgG2 (present in 8 samples) or MOG-IgG4 (weakly positive in two samples) (see Table 1 for details); MOG-IgG2 was present together with IgG1 in four samples and together with both IgG1 and IgG3 in four samples.

As mentioned above, all patients, including those with exclusive or predominant IgG3 reactivity in the follow-up sample, had previously tested positive for MOG antibodies at least once with standard, i.e. not subclass-specific, assays, including live CBA in all but two cases (see Table 1, last column, for details), corroborating the validity of the subclass-specific test results. Of these patients, 21 had been positive for MOG-IgG and 1 for MOG-IgA in one or more previous samples (including in two or more different assays in at least 18 of them; see last column of the Table 1 for details).

The present findings are of high potential clinical relevance: (1) Some of the currently most widely used fixed and live routine assays assessing total MOG-IgG might miss some patients who are exclusively or predominantly positive for MOG-IgG3. (2) In a recent proposal for new diagnostic criteria for MOG-EM/MOGAD [2], the use of IgG1-specific assays was advocated as an alternative to Fc-specific assays [2]. Indeed, most patients with MOG-IgG are predominantly MOG-IgG1-positive [16]. However, our data suggest that the use of IgG1-specific assays only might result in overlooking rare patients who are exclusively MOG-IgG3-positive (either from the beginning or due to ‘partial [i.e. subclass-restricted or -accentuated] seroreversion’, either spontaneous or treatment-induced, later in the disease course), or cause diagnostic issues, since MOG-IgG1 signals were very weak in several of the clearly MOG-IgG3 positive samples. Additional testing by means of IgG3-specific assays could thus improve MOG-IgG serology and, in consequence, affect the management of MOG-EM/MOGAD.

Given that the only patient in our cohort who had previously been diagnosed with MOG-IgA^+^/MOG-IgG^–^ MOG-EM/MOGAD, a new recently described subvariant [1], turned out to be strongly positive for MOG-IgG3 and weakly positive for MOG-IgG1, -IgG2, and IgG4, the use of IgG subclass-specific assays might be advisable before a diagnosis of MOG-IgA^+^/MOG-IgG^–^ MOG-EM/MOGAD is made. Of note, this patient was also weakly positive for total MOG-IgG in a fixed CBA at our institution, but the signal obtained in the IgG3-specific assays was much stronger (Table 1). MOG-IgA and MOG-IgM were negative in all of the other patients tested (Table 1).

Interestingly, two follow-up samples were available from one of the previously MOG-IgG-positive patients (no. 8; previous MOG-IgG titre 1:5,120 in a live CBA; positive also in a fixed CBA). While the first follow-up sample had turned negative for total MOG-IgG in a fixed CBA and in 2 of 3 live CBAs but remained positive for MOG-IgG3 (and MOG-IgG1), the second follow-up sample was negative also for MOG-IgG3 (but not MOG-IgG1), demonstrating that ‘partial seroreversion’ may also occur in the other direction, resulting in selective MOG-IgG1 positivity (Table 1). Moreover, MOG-IgG3 was negative also in several other samples that were positive for total MOG-IgG and/or MOG-IgG1 (Table 1). Exclusive testing for MOG-IgG3 is, therefore, discouraged. However, supplementary testing, either in parallel or as a second step, for MOG-IgG3 may be a highly useful addition to MOG-IgG1 testing and help to avoid false-negative results.

Due to the chromosomal order of the respective genes (VDJ-γ3-γ1-γ2-γ4), individual antibodies may switch from IgG3 to IgG1 but not vice versa. However, MOG-IgG3 antibodies may theoretically nonetheless (re)appear in patients previously positive exclusively for MOG-IgG1 at one point in time, namely if MOG-IgG3 antibodies originating from other clones that transiently fell below the cut-off rise again or if additional B cells clones recognizing other MOG epitopes get de novo-activated later in the course of disease.

Altogether, these findings highlight that there might be a dynamic evolution of MOG antibody subclasses during the course of the disease, which consecutively renders MOG antibody testing more complex than previously thought.

This said, it should be emphasized that the currently used routine assays are all thought to be highly sensitive in the majority of patients [6, 28]. When it comes to low titre samples, however, discordance between assays has been noted. In this subgroup of patients, additional testing for MOG-IgG3 (and MOG-IgG1) may be of particular value.

In contrast to (typically polysaccharide-specific) IgG2, which has been previously described as the dominant subclass of MOG-IgG in single patients with suspected MOG-EM/MOGAD [6], IgG3 (which mainly recognizes proteins) is a strong complement activator (even stronger than IgG1). This is important, since MOG-IgG has been shown to cause complement-mediated demyelination [27, 29]. Moreover, IgG3 has the capacity to bind to all major FcR types (with significant allotype-specific variation existing [4]) and is particularly effective in the induction of effector functions (e.g. stronger binding to Fcγ receptors [FcγR] I and II, which are present, for instance, on monocytes, macrophages, neutrophils and dendritic cells, than IgG1) [31]. MOG-IgG3 may thus well be involved in the immunopathogenesis of MOG-EM/MOGAD in addition to MOG-IgG1, and MOG-IgG3 seropositivity in patients with MOG-EM/MOGAD might therefore also have therapeutic implications. Of note, rozanolixizumab, a monoclonal antibody blocking the neonatal Fc receptor, which has already been approved for the treatment of myasthenia gravis (MG) and is currently being investigated in a phase-III study in MOG-EM/MOGAD, has been reported to preferentially lower IgG3 serum levels [21]. It will be of interest to investigate whether MOG-IgG3 antibodies recognize distinct epitopes, as has been reported for neurofascin-IgG3 [30].

It is unknown why some patient sera exclusively or predominantly harbour MOG antibodies of the IgG3 subclass. Notably, the first sample from our index patient was taken less than 3 months after plasma exchange (PLEX) and around 1 month after the first cycle of rituximab. While PLEX seems to remove all subclasses to a similar extent [26], a recent study on PLEX in patients with MG showed that return to baseline autoantibody titres was fastest for AChR-IgG3, which had recovered at week 3, while AChR-IgG1 had reached 20% of baseline by that time (in line with the increased fractional turnover of IgG3 also in healthy individuals) [7]. It is conceivable that the same might apply to MOG-IgG. Whether rituximab differentially affects the four IgG subclasses is unknown and the topic of current investigation. However, even if treatment was responsible for the predominance of IgG3 in our patient, this would not diminish the relevance of our findings. Indeed, PLEX and rituximab are standard treatments both in patients with MOG-EM/MOGAD and in patients with syndromes considered to pose a high risk of MOG-EM/MOGAD, such as “neuromyelitis optica spectrum disorders without AQP4-IgG” [8, 33] or chronic relapsing inflammatory optic neuropathy (CRION), meaning that serological testing may be influenced by those treatments in many patients. No treatment data are currently available for the remaining patients of this cohort, but this will be addressed in future studies.

Of note, IgG3 has also been reported to be the first IgG subclass to appear in patients with viral infection [31]. Viral infections have been shown to precede MOG-EM/MOGAD attacks, both at disease onset and later in the course, in many cases [14]. Moreover, ADEM, which not uncommonly occurs in a para-/postinfectious setting [25], has been shown to be associated with MOG-IgG in a substantial proportion of cases. It is therefore tantalizing to speculate that predominant MOG-IgG3 seropositivity might thus denote a para-/postinfectious aetiology of acute attacks in MOG-EM/MOGAD. Studies on this question are currently ongoing at our institutions. By contrast, chronic infections can result in reduced total IgG3 concentrations [3].

IgG3 antibodies have also been reported in other neurological antibody-associated diseases, including in MG (with ligand site-binding antibodies being selectively of the IgG3 subclass in some patients [22]), and predominant IgG3-seropositivity has been described in primary biliary cirrhosis (PBC) as well as in thrombocytopenic/haemolytic autoimmune conditions [31]. Moreover, total IgG3 serum levels were significantly higher in a large study in PBC, systemic lupus erythematosus, primary Sjögren’s syndrome, and systemic sclerosis than in healthy controls [34]. In anti-neurofascin syndrome, IgG3 autoantibodies have been reported to define a particularly severe clinical phenotype [30].

The reason for the higher sensitivity of IgG3-specific testing than of assays using H+L- or Fc/Fcγ-specific secondary antibodies, as observed in some of our patients, is unknown, but imbalanced reactivity of some of the currently used H+L- or Fc/Fcγ-specific detection antibodies towards the four human IgG subclasses, a phenomenon well-known for anti-rodent IgG antibodies used in other applications, may be involved. Indeed, IgG3 is structurally unique by virtue of its extended hinge regions, additional inter-heavy chain disulfide bonds and additional glycosylation sites (Figure 1) [31]. Moreover, IgG3 differs from the other subclasses by an excessive number of known allotypes/polymorphisms, which vary with regard to amino acid sequences, number and type of hinge region exons and hinge length, flexibility, and relative spatial orientation of the Fab and Fc regions in bound IgG3 (Figure 1) [31]. Some of these differences could well compromise binding of non-IgG3-specific detection antibodies in serological assays.

Our findings likewise imply that other cell-based assays for detecting anti-neural autoantibodies that use the same (or same class of) detection antibodies may be affected by similar issues, provided IgG3 autoantibodies play a role in those diseases. We are currently investigating this in aquaporin-4-IgG-positive NMOSD and various forms of autoimmune encephalitis.

It is tantalizing to speculate that the diagnostic sensitivity of MOG-IgG assays could be increased using a mixture of IgG1- and IgG3-specific detection antibodies, as such an approach may additively result in stronger overall signal intensity. Interestingly, however, most patients in our cohort showed either strong IgG3 reactivity with weak or absent IgG1 reactivity or strong IgG1 with weak or absent IgG3 reactivity (Table 1), raising the question of whether predominant IgG3 and IgG1 reactivity might even denote different clinical, pathogenetic or prognostic subsets (similar to what has been suggested in anti-neurofascin syndrome) [30]. Alternatively, they may represent different disease stages or reflect different treatments, as discussed above. However, insufficient data were available retrospectively to address this question in the present cohort.

It is of pathophysiological interest that some IgG3 allotypes are associated with a shorter half-life (7 days vs. 21 days); some IgG3 allotypes may affect C1q complement binding; Ig allotypes also differ with respect to Fcγ receptor binding efficacy and thus antibody-dependent cell-mediated cytotoxicity; finally, absolute IgG concentrations depend on allotypes [31]. Not only the exact subclass composition of total MOG-IgG but also IgG allotypes may thus affect disease severity in MOG-EM/MOGAD. For our understanding of differences observed between studies from different regions of the world, it is of potential relevance that allelic differences have been observed not only between individuals but also between populations [31].

MOG-IgG3 antibodies have previously been reported in three patients with negative results for total MOG-IgG, as determined by a non-subclass-specific assay, in one study [24]; in three out of 15 patients with discordant results from three different non-subclass-specific CBAs in a second study [6]; and, at high titre (1:3200), in a single patient with ON and myelitis with patchy cord lesions [32]. However, in contrast to the present study, no unequivocally MOG-IgG-positive earlier or follow-up samples from the same patients that would corroborate the specificity of those findings were available. Moreover, MOG-IgG3 titres were below the cut-off commonly applied in that type of CBA in one of these studies [24] and no healthy controls were included in any of the three previous studies [6, 24, 32].

While we consider the fact that all samples were taken from patients with a previous diagnosis of MOG-EM/MOGAD (based on unequivocally positive MOG-IgG test results from previous samples and typical clinicoradiological findings) a particular strength of this study, we count the retrospective nature of this report and the limited treatment data available among its potential limitations. We can also not completely rule out the possibility that our study actually underestimates the frequency of MOG-IgG3 in MOG-EM/MOGAD: IgG is generally considered to be highly stable and serum samples are thus tested (or stored) for MOG-IgG and other autoantibodies without further pre-treatment as the standard procedure at most laboratories, including for this study. However, IgG3 is thought to be more susceptible to proteolytic degradation than other IgG subclasses owing to the long hinge region. The use of aprotinin or other inhibitors of proteolysis might thus be advisable in future studies.

We conclude that testing for IgG3 may, in some cases, improve the detection of MOG-IgG in serum samples. Our findings are in line with previous studies that suggested slightly higher sensitivity of subclass-specific assays than of (at least some) H+L- or Fc-specific assays, but suggest that IgG1-subclass-restricted assays (as currently performed at some institutions) may miss some MOG-IgG-positive samples. Considering that IgG1 is present in the majority of patients, MOG-IgG3 testing could be performed as a subsidiary diagnostic step for reasons of test economy, i.e. only if total MOG-IgG and MOG-IgG1 are negative or present only at low or borderline titre. MOG-IgG3-specific testing may also be considered before a decision is made to stop treatment in patients with MOG-EM/MOGAD due to reversion to (suspected) seronegativity. Testing for MOG-IgG3 in samples already shown to be unequivocally positive for total MOG-IgG or for MOG-IgG1 seems currently dispensable from a diagnostic point of view but may become relevant if future studies should reveal therapeutic or prognostic implications of MOG-IgG3 (co-)seropositivity. More studies are needed to better understand the pathogenetic, diagnostic and prognostic roles and the serum dynamics of the various MOG-IgG subclasses in patients with MOG-EM/MOGAD. Our study also provides a rationale for studies investigating the role of IgG3 antibodies in other inflammatory disorders of the central nervous system, such as NMOSD or autoimmune encephalitis.

### Supplementary Information

Below is the link to the electronic supplementary material.

Supplementary file1 (DOCX 3293 KB—Supplementary Figure 1 Binding of immunoglobulin (Ig) 1 (first column), IgG2 (second column), IgG3 (third column) and IgG4 (last column) in two serum samples from the index patient (no. 1A and 1B in the Table 1), obtained at an interval of 2 years, to HEK293 cells transfected with full-length human MOG (A, C) and to empty vector-transfected control cells (B, D), as detected by use of IgG subclass-specific secondary antibodies labelled with AF568 (red) (see ref. [15] for methods). Cell nuclei were counterstained with DAPI (blue). Both samples show strong binding of IgG3 to the MOG-transfected cells (baseline: A3; after 2 years: C3) but not to the control cells (B3, D3). By contrast, only very weak MOG binding of IgG1 was noted at baseline (A1; inset: same sample with reduced DAPI background staining) and no unequivocal binding after 2 years (C1); again, no binding to the control cells was discernible (B1, D1). Very weak binding of IgG2 (baseline: A2; after 2 years: C2) and IgG4 (A4) to the MOG-transfected cells, but not to the control cells (B2, D2 and B4), was observed. Note that the DAPI (blue) channel is not shown in either panels A2 and B2 or panels A4 and B4 to improve the visibility of the weak IgG2 and IgG4 signal, respectively (red). See rows 1 and 2 in the Table 1 for more details on these samples. Abbreviations: AF, AlexaFluor®; DAPI, 4′, 6-diamidino-2-phenylindole; HEK293, human embryonic kidney 293 cells; MOG, myelin oligodendrocyte glycoprotein)

## Data Availability

All data are available within the paper and the supplementary material

## Abbreviations

AChR: Acetylcholine receptor
AQP4: Aquaporin-4
CBA: Cell-based assay
CNS: Central nervous system
Fc: Crystallizable fragment
IgG: Immunoglobulin G
H+L: Heavy and light chain
MG: Myasthenia gravis
MOG: Myelin oligodendrocyte glycoprotein
MOG-EM/MOGAD: MOG antibody-associated encephalomyelitis/MOG antibody-associated disease
NMOSD: Neuromyelitis optica spectrum disorders
PBC: Primary biliary cirrhosis
PLEX: Plasma exchange
RTX: Rituximab
