# Supplementary material for: Improving the sensitivity of myelin oligodendrocyte glycoprotein-antibody testing: exclusive or predominant MOG-IgG3 seropositivity—a potential diagnostic pitfall in patients with MOG-EM/MOGAD
Source: J Neurol. 2024 Apr 13;271(7):4660–71. doi: 10.1007/s00415-024-12285-5 (PMC11233316; doi:10.1007/s00415-024-12285-5)
Supplement: Supplementary file 1 — Supplementary file1 (DOCX 3293 KB—Supplementary Figure 1 Binding of immunoglobulin (Ig) 1 (first column), IgG2 (second column), IgG3 (third column) and IgG4 (last column) in two serum samples from the index patient (no. 1A and 1B in the Table 1), obtained at an interval of 2 years, to HEK293 cells transfected with full-length human MOG (A, C) and to empty vector-transfected control cells (B, D), as detected by use of IgG subclass-specific secondary antibodies labelled with AF568 (red) (see ref. [15] for methods). Cell nuclei were counterstained with DAPI (blue). Both samples show strong binding of IgG3 to the MOG-transfected cells (baseline: A3; after 2 years: C3) but not to the control cells (B3, D3). By contrast, only very weak MOG binding of IgG1 was noted at baseline (A1; inset: same sample with reduced DAPI background staining) and no unequivocal binding after 2 years (C1); again, no binding to the control cells was discernible (B1, D1). Very weak binding of IgG2 (baseline: A2; after 2 years: C2) and IgG4 (A4) to the MOG-transfected cells, but not to the control cells (B2, D2 and B4), was observed. Note that the DAPI (blue) channel is not shown in either panels A2 and B2 or panels A4 and B4 to improve the visibility of the weak IgG2 and IgG4 signal, respectively (red). See rows 1 and 2 in the Table 1 for more details on these samples. Abbreviations: AF, AlexaFluor®; DAPI, 4′, 6-diamidino-2-phenylindole; HEK293, human embryonic kidney 293 cells; MOG, myelin oligodendrocyte glycoprotein) [file 415_2024_12285_MOESM1_ESM.docx]

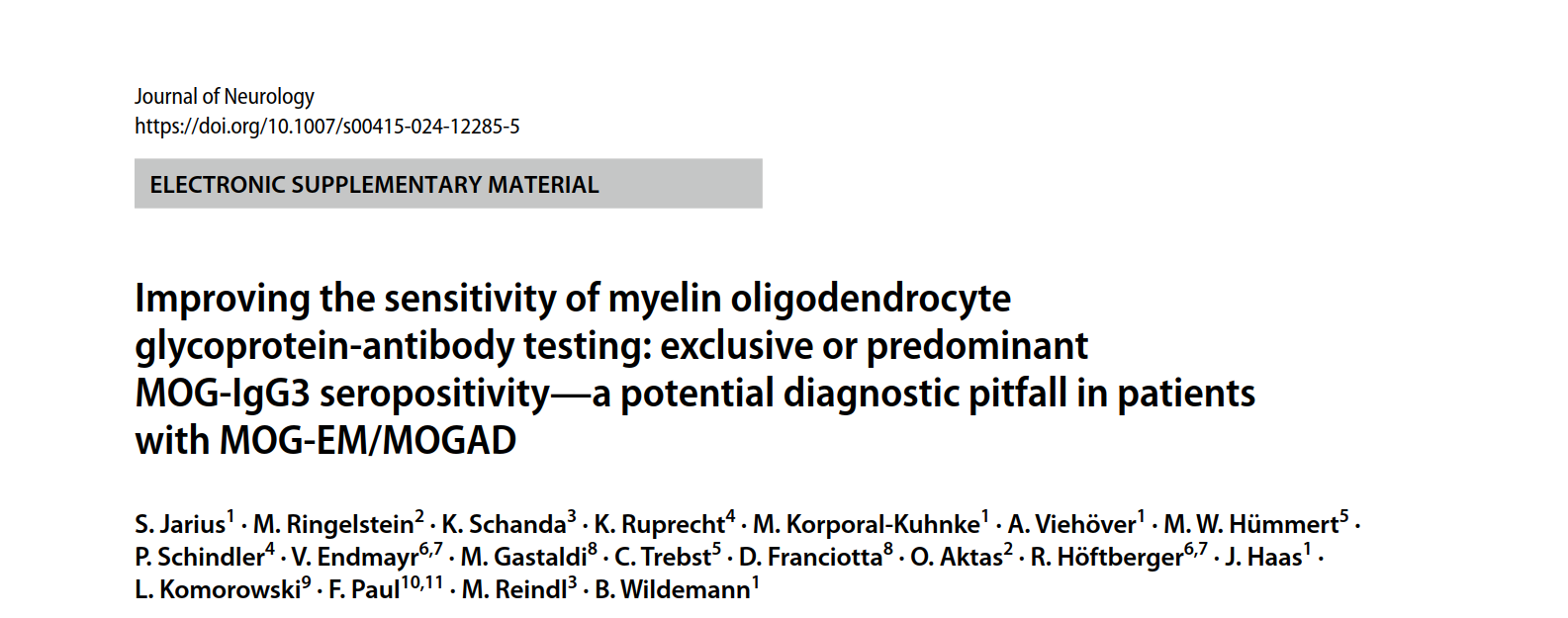

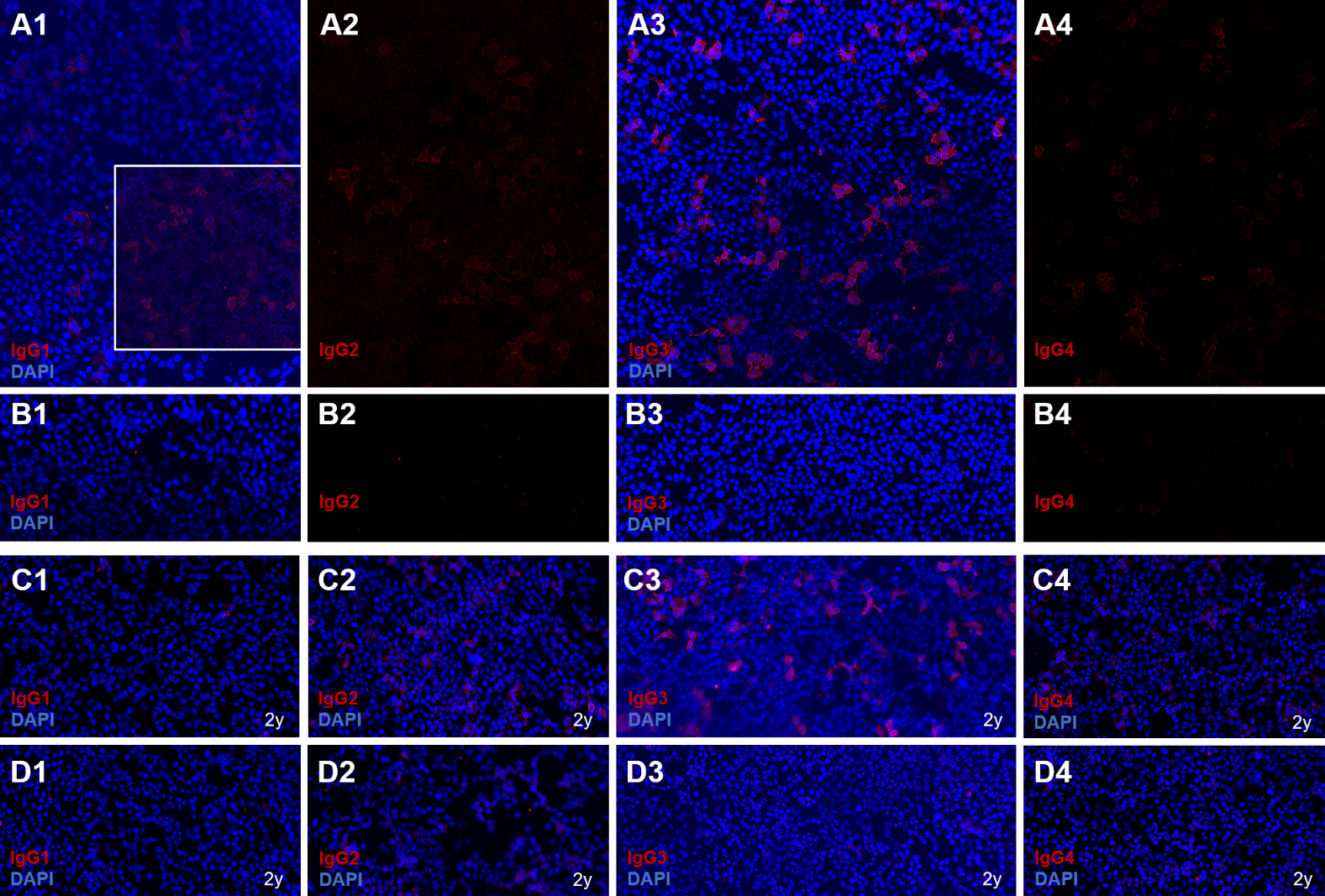


Supplementary Figure. Binding of immunoglobulin (Ig) 1 (first column), IgG2 (second column), IgG3 (third column) and IgG4 (last column) in two serum samples from the index patient (no. 1A and 1B in the Table), obtained at an interval of 2 years, to HEK293 cells transfected with full-length human MOG (A, C) and to empty vector-transfected control cells (B, D), as detected by use of IgG subclass-specific sheep anti-human IgG secondary antibodies and donkey anti-sheep IgG tertiary antibodies labelled with AF568 (red) (see ref. [15] for methods). Cell nuclei were counterstained with DAPI (blue). Both samples showed strong binding of IgG3 to the MOG-transfected cells (baseline: A3; after 2 years: C3) but not to the control cells (B3, D3). By contrast, only very weak MOG binding of IgG1 was noted at baseline (A1; inset: same sample with reduced DAPI background staining) and no unequivocal binding after 2 years (C1); again, no binding to the control cells was discernible (B1, D1). Very weak binding of IgG2 (baseline: A2; after two years: C2) and IgG4 (A4) to the MOG-transfected cells, but not to the control cells (B2, D2 and B4, D4), was observed. Note that the DAPI (blue) channel is not shown in either panels A2 and B2 or panels A4 and B4 to improve the visibility of the weak IgG2 and IgG4 signal, respectively (red). See rows 1 and 2 in the Table for more details on these samples. Abbreviations: *AF* AlexaFluor®; *DAPI* 4′,6-diamidino-2-phenylindole; *HEK293* human embryonic kidney 293 cells; *MOG* myelin oligodendrocyte glycoprotein.
